# Supplementary material for: EphrinA5 regulates cell motility by modulating Snhg15/DNA triplex-dependent targeting of DNMT1 to the Ncam1 promoter
Source: Epigenetics Chromatin. 2023 Oct 26;16:42. doi: 10.1186/s13072-023-00516-4 (PMC10601256; doi:10.1186/s13072-023-00516-4)
Supplement: Supplementary file 1 — Additional file 1: Figure S1. Western blots confirming the specificity of antibodies against DNMT1 and EZH2. (a) Immunoprecipitations from N2a whole cell lysate with normal rabbit IgG (#12–370, Merck) and rabbit anti-DNMT1 (#70–201, BioAcademia) antibodies. Detection was performed with the mouse anti-DNMT1 antibody (1:250, # ab13537, abcam). (b) The EZH2 antibody (1:1000, #5246, Cell Signaling Technology) was used for detection on whole cell lysate from the murine medial ganglionic eminence (MGE). Protein sizes are indicated in kDa, identified through SERVA Protein standard III. Figure S2. CLIP revealed no interaction of Snhg15 and EZH2 in CB cells. RNA recovery for IgG and anti-EZH2 antibody CLIP samples in CB cells (N = 5 biological replicates). For all investigated amplicons, the recovery for EZH2 could not be statistically differentiated from the IgG-based pulldown. Whiskers of the box plots extend 1.5 times the interquartile range from the 25th and 75th percentiles (Tukey style) while outliers are represented by hollow dots. Significances were determined with two-tailed Student’s t-test. ctrl-Fc: control Fc, efnA5-Fc: ephrinA5-Fc, CB: cerebellar granule, CLIP: UV cross-linking and immunoprecipitation. Figure S3. Expression of NCAM1/Ncam1 is implicated in low-grade glioma as well as the migration of CB cells, where it can be downregulated via RNA silencing. (a) High expression levels of NCAM1 are associated with increased patient survival in low-grade glioma. Survival analysis is based on clinical data and gene expression counts from tumor samples of lower grade glioma patients downloaded from BioPortal (http://www.cbioportal.org/study/clinicalData?id=lgg_tcga) and The Cancer Genome Atlas (TCGA), respectively. (b) Knockdown efficiency of the applied Ncam1 siRNA (N = 3 biological replicates). (c) Quantitative analysis of migration distance of CB cells (n = 557 for ctrl siR + ctrl-Fc, n = 455 for ctrl siR + efnA5-Fc, n = 481 for Ncam1 siR + ctrl-Fc, n = 495 for Nc [file 13072_2023_516_MOESM1_ESM.pdf]

# Supplementary Figures

Yildiz et al.

# Supplementary Figure 1

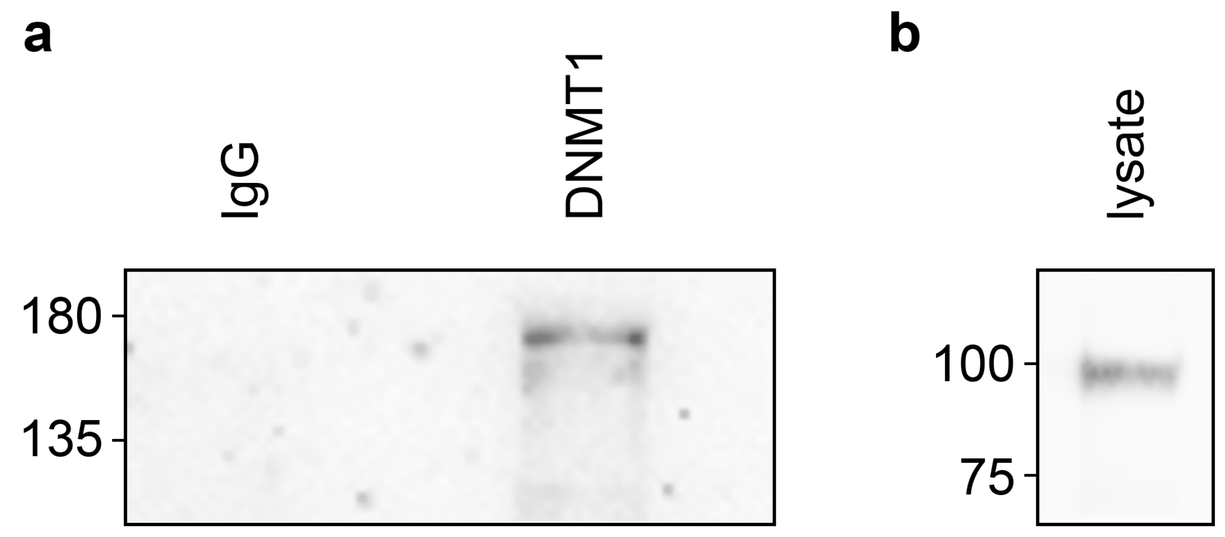

# Supplementary Figure 2

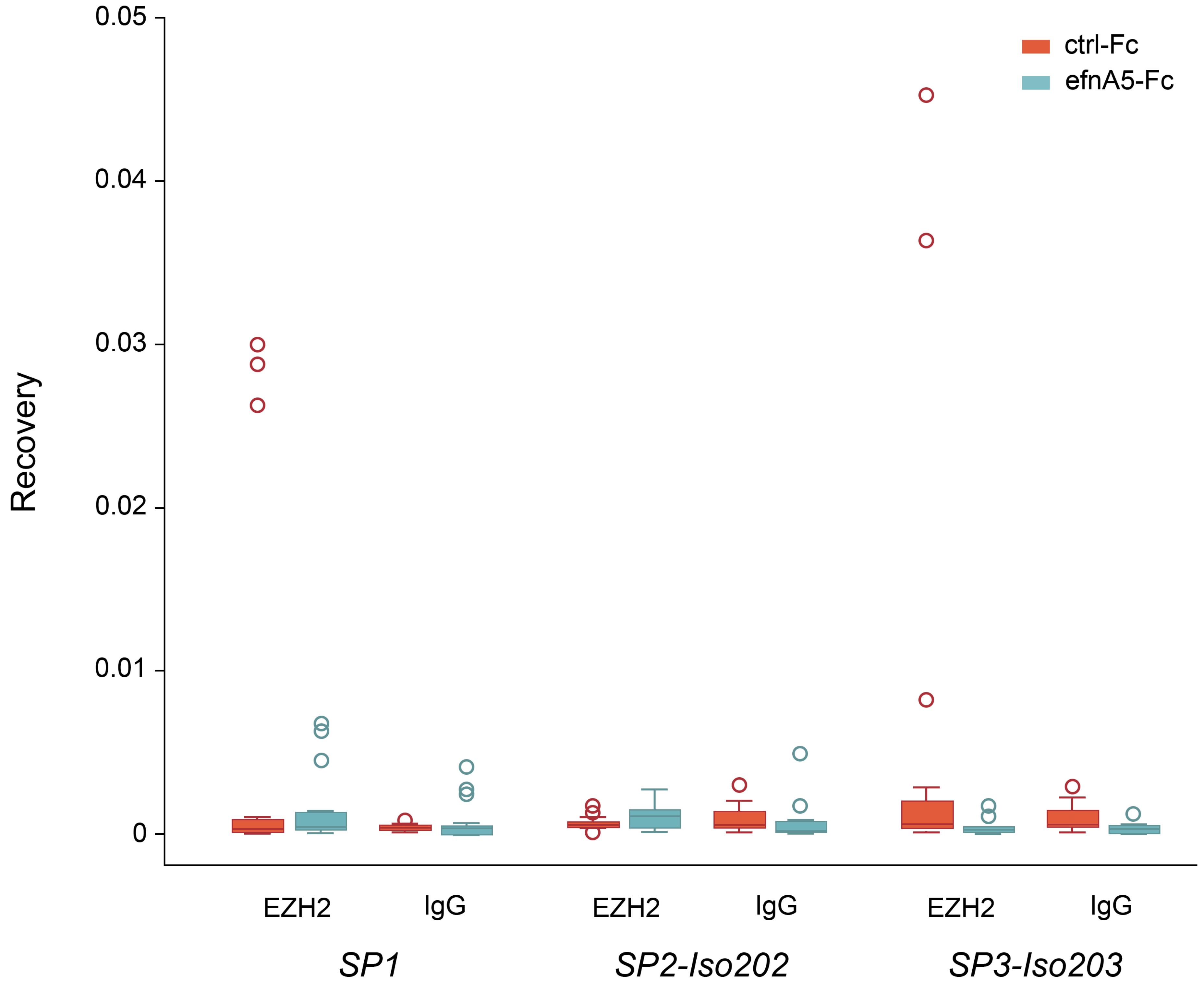

# Supplementary Figure 3

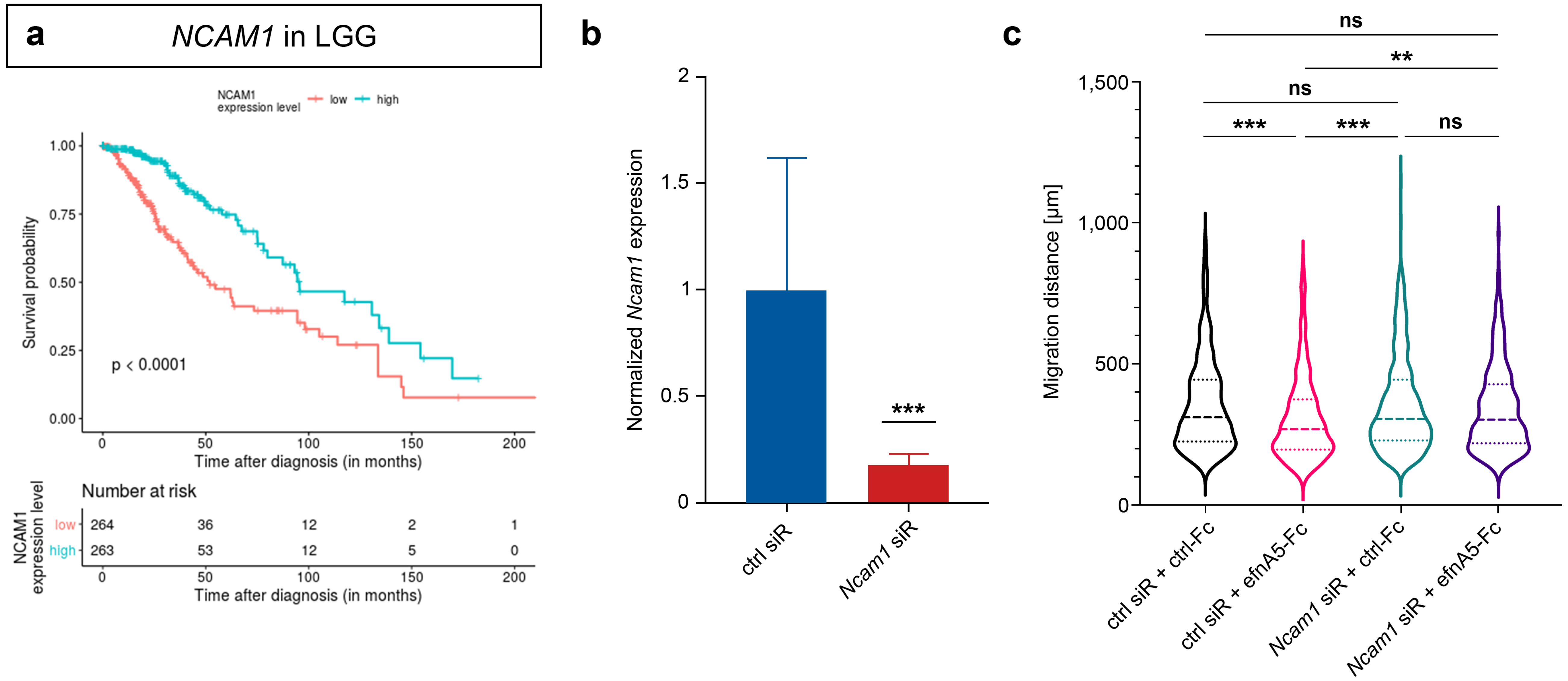

# Supplementary Figure 4

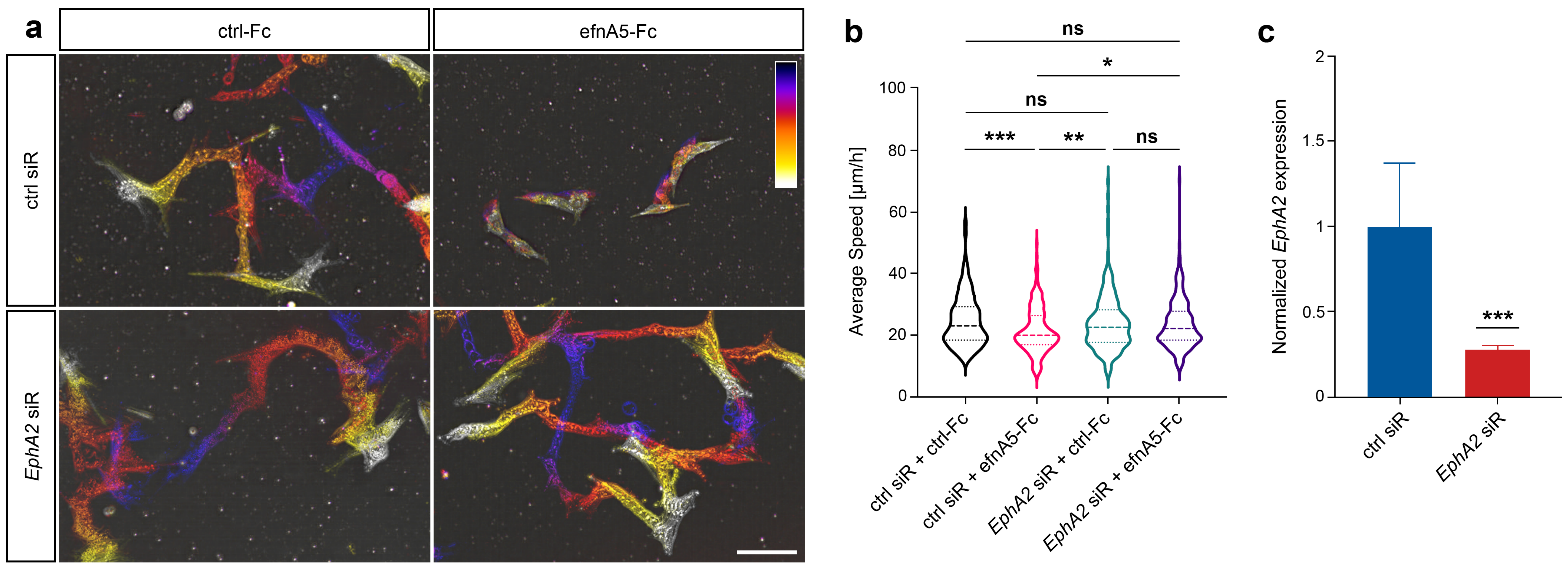

# Supplementary Figure 5

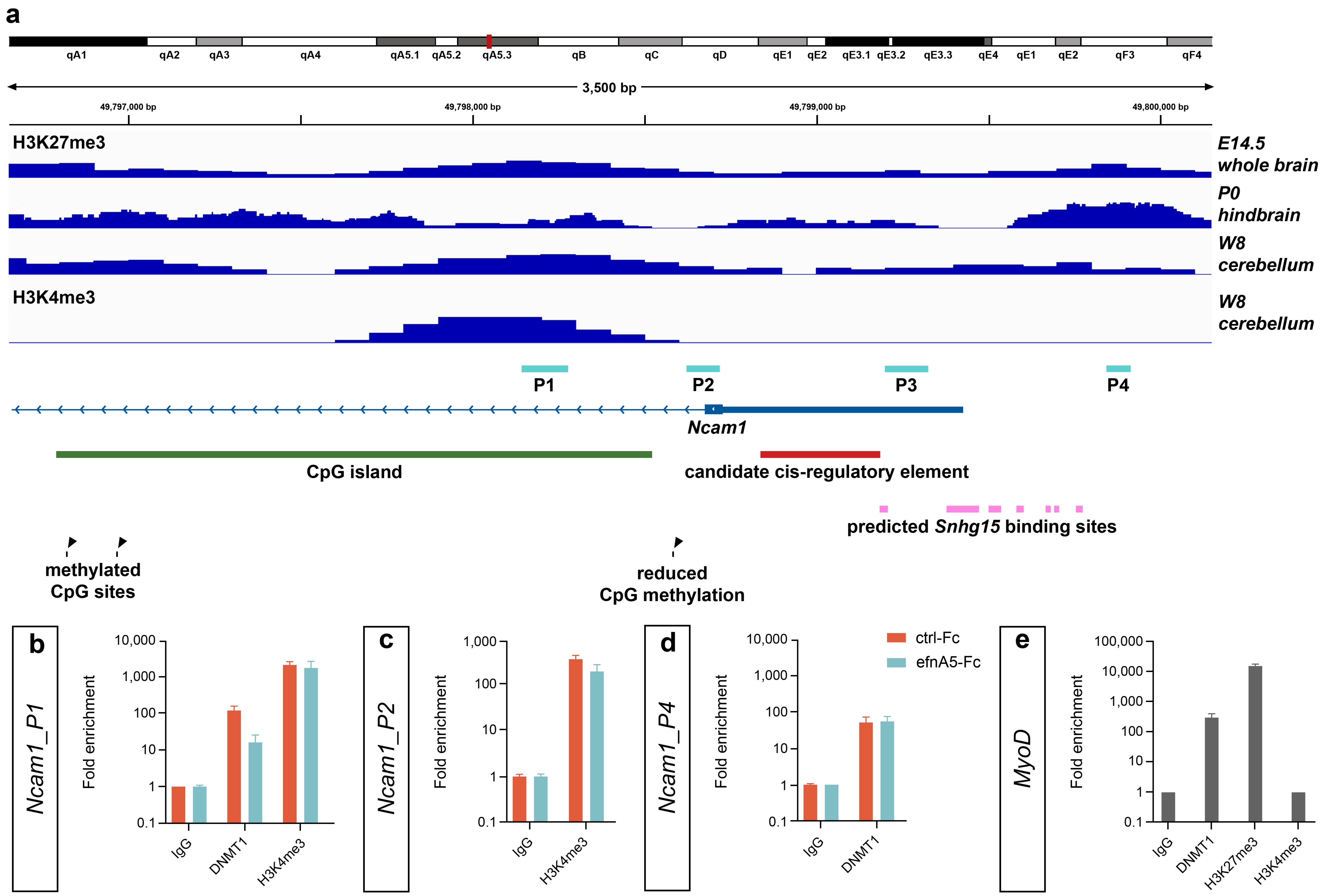

# Supplementary Figure 6

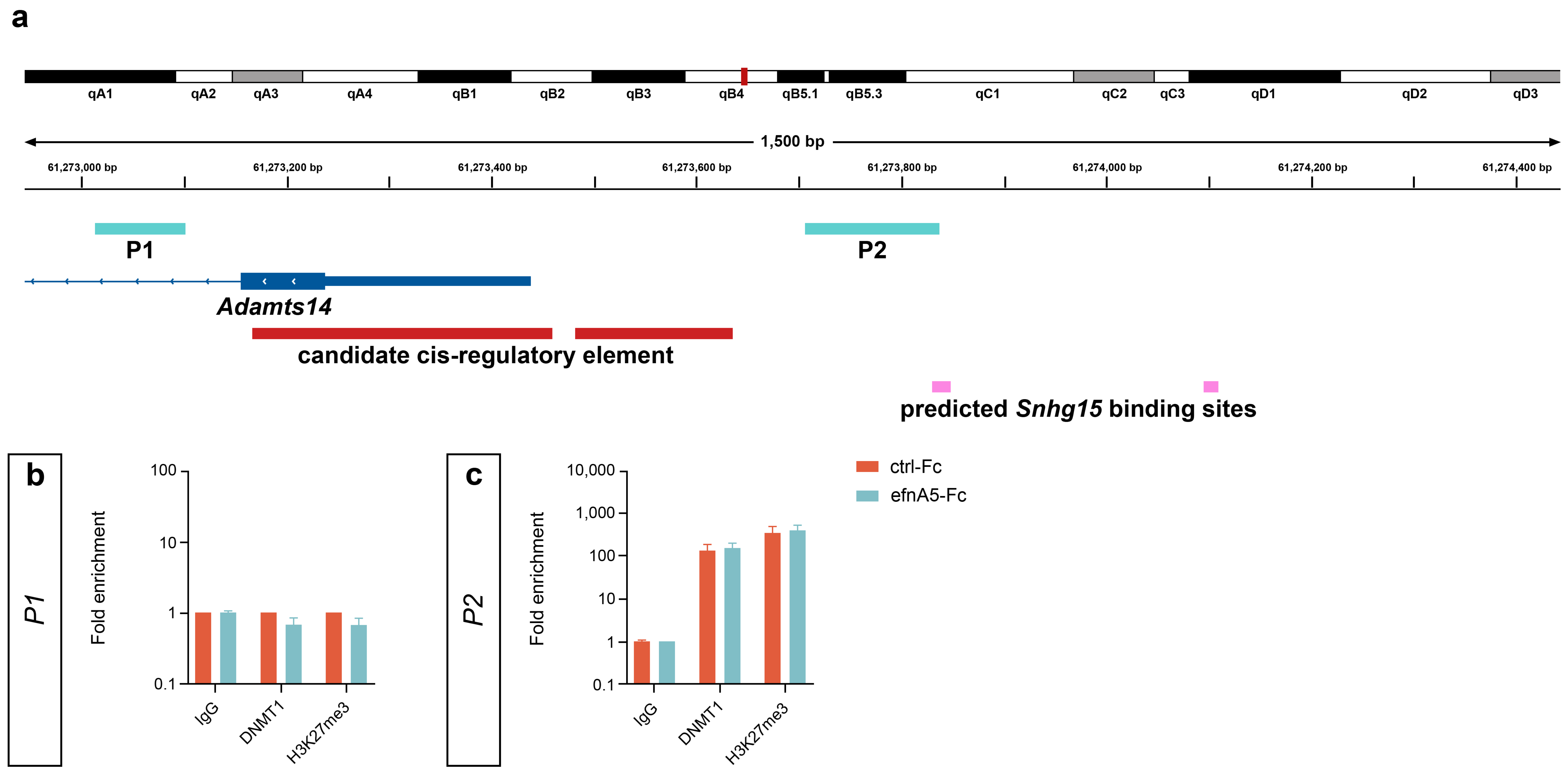

# Supplementary Figure 7

**a**

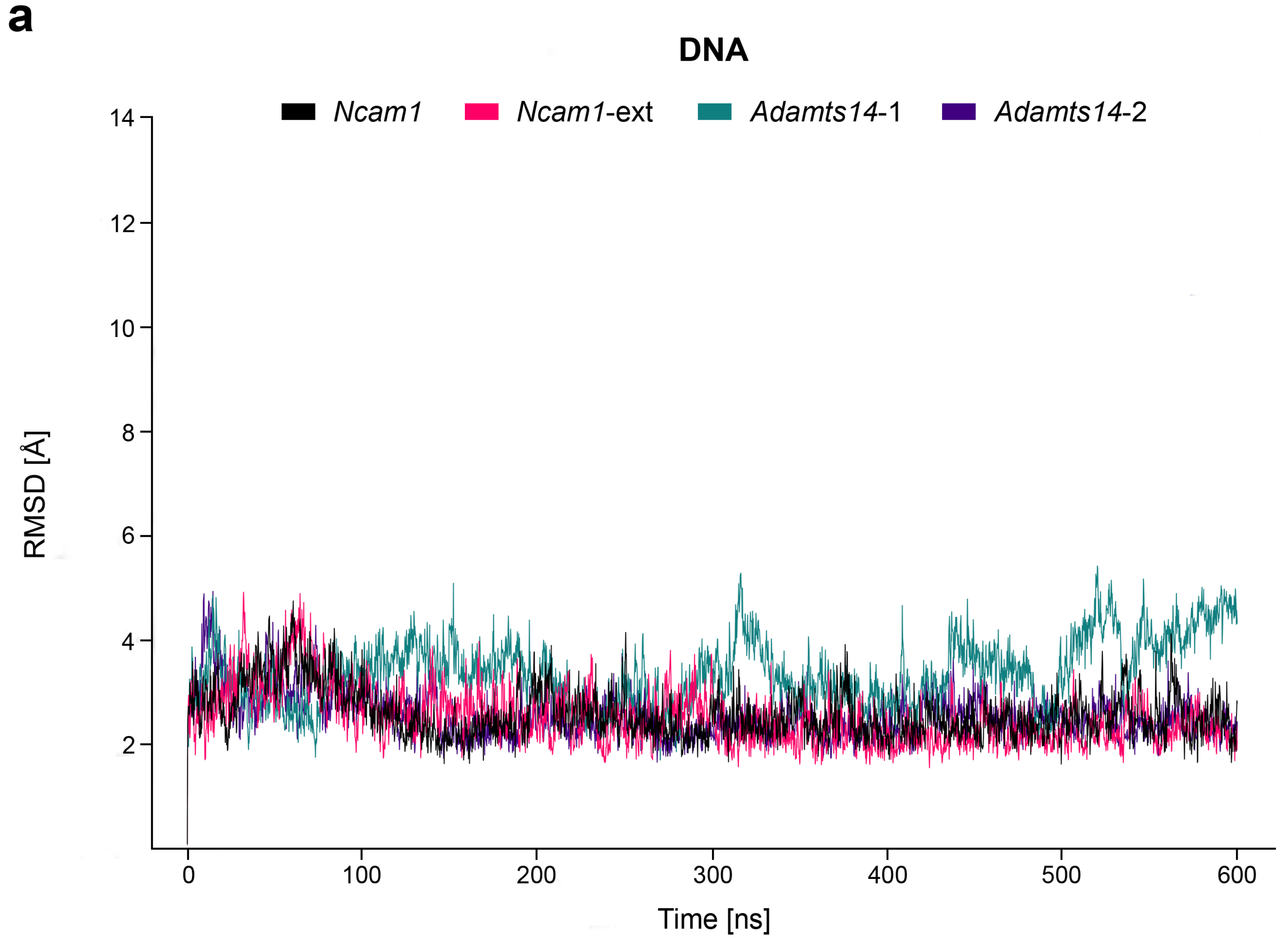


**b**

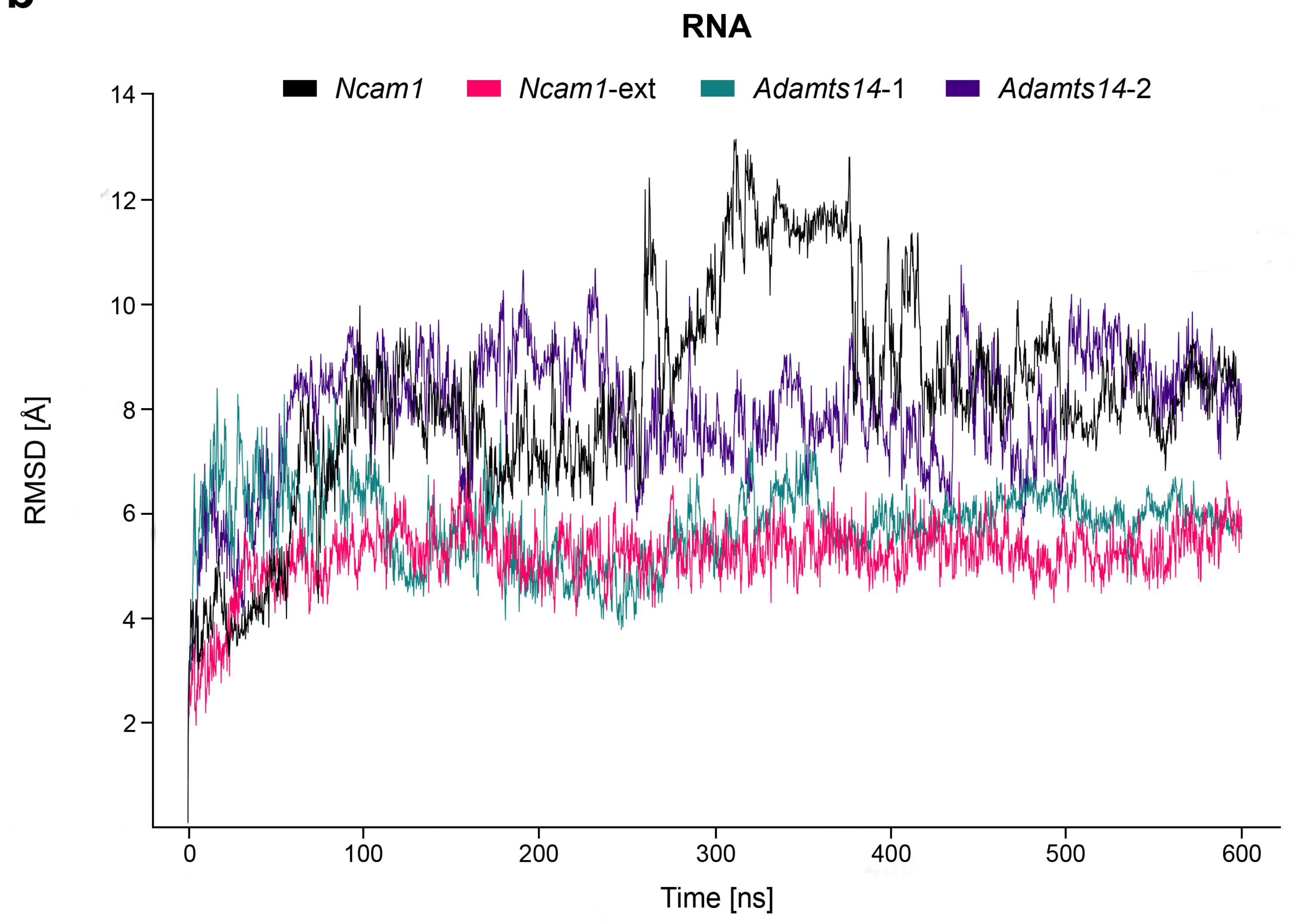

# Supplementary Figure 8

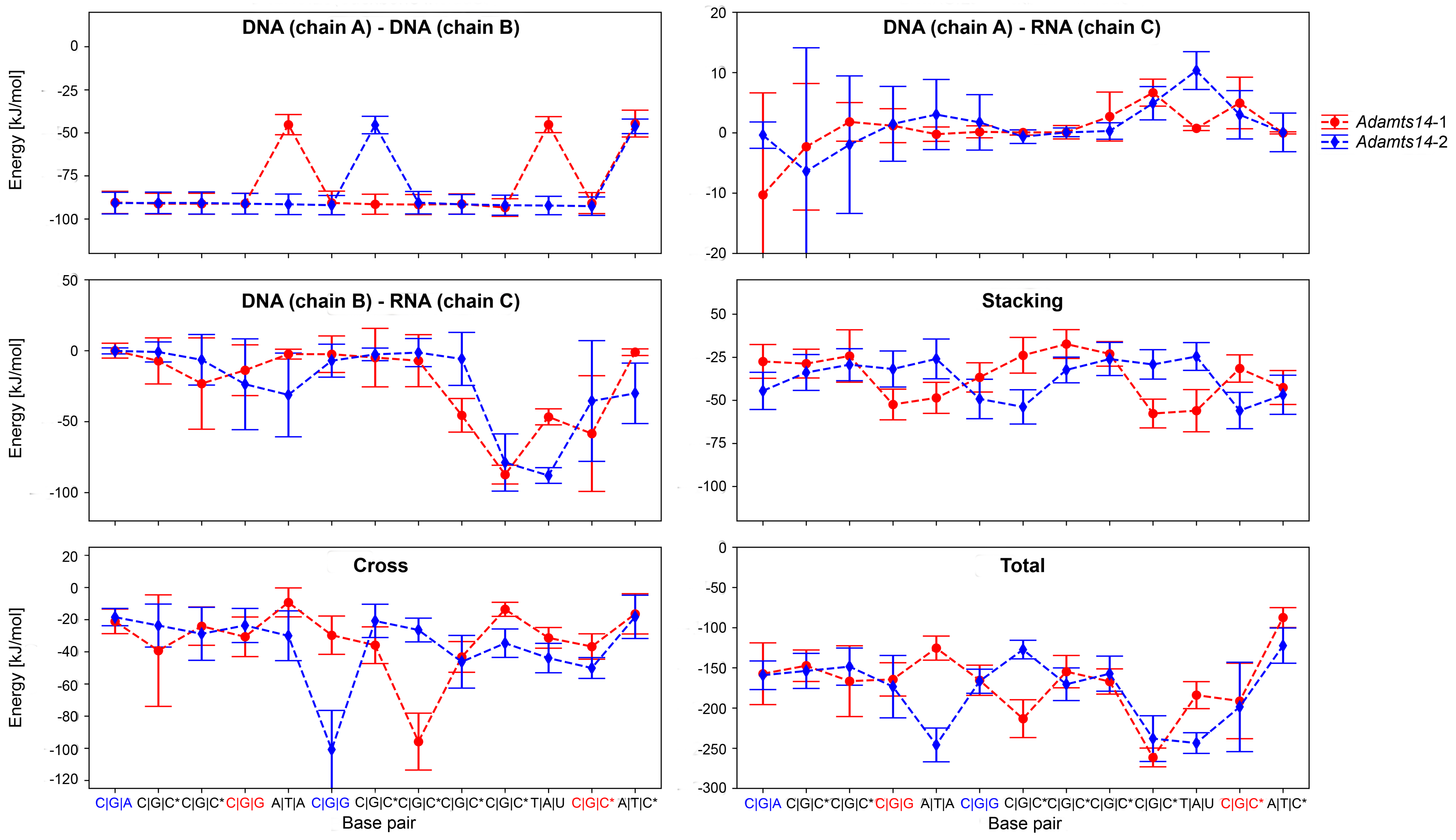

# Supplementary Figure 9

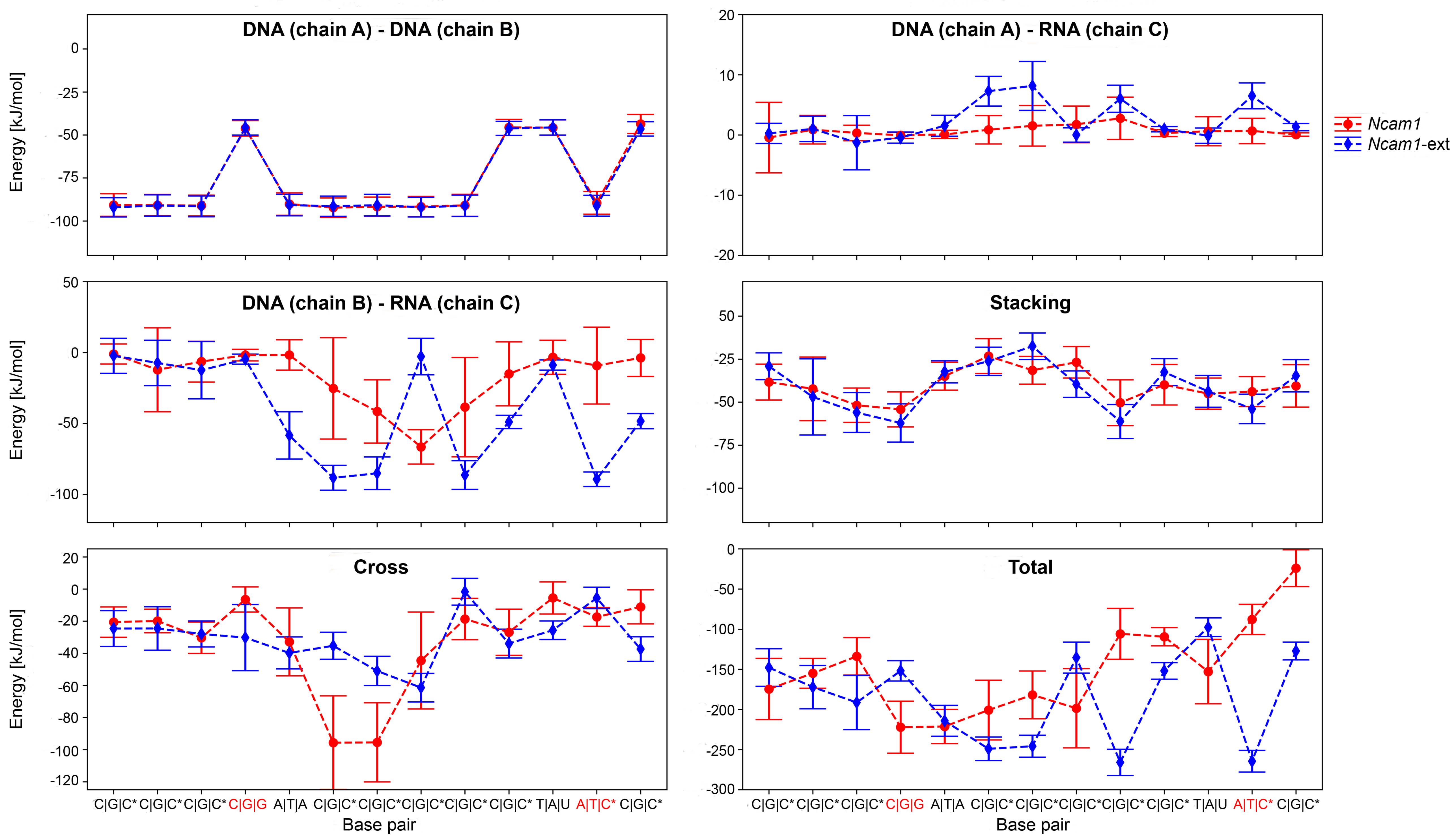

# Supplementary Figure 10

**a**

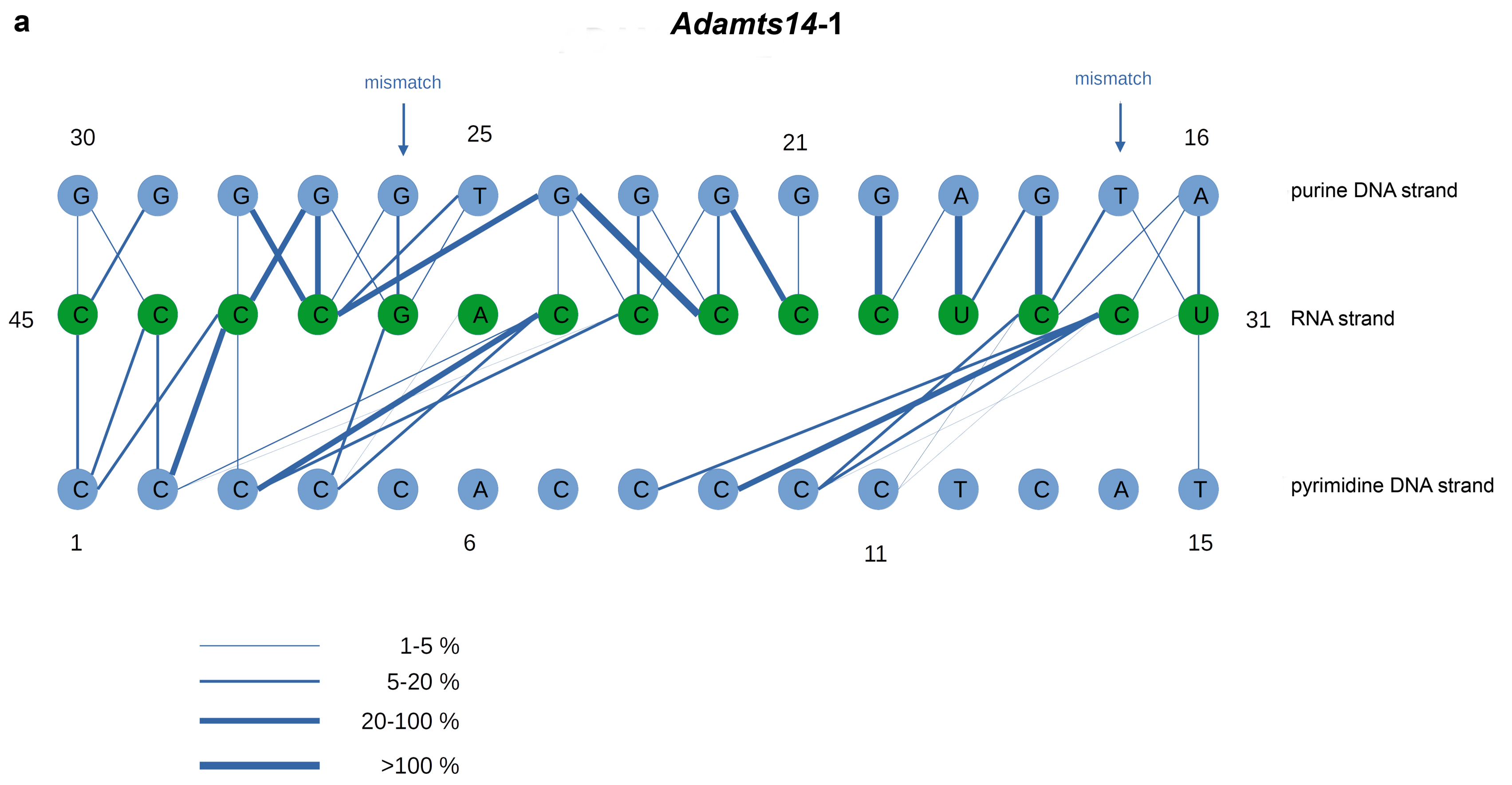


**b**

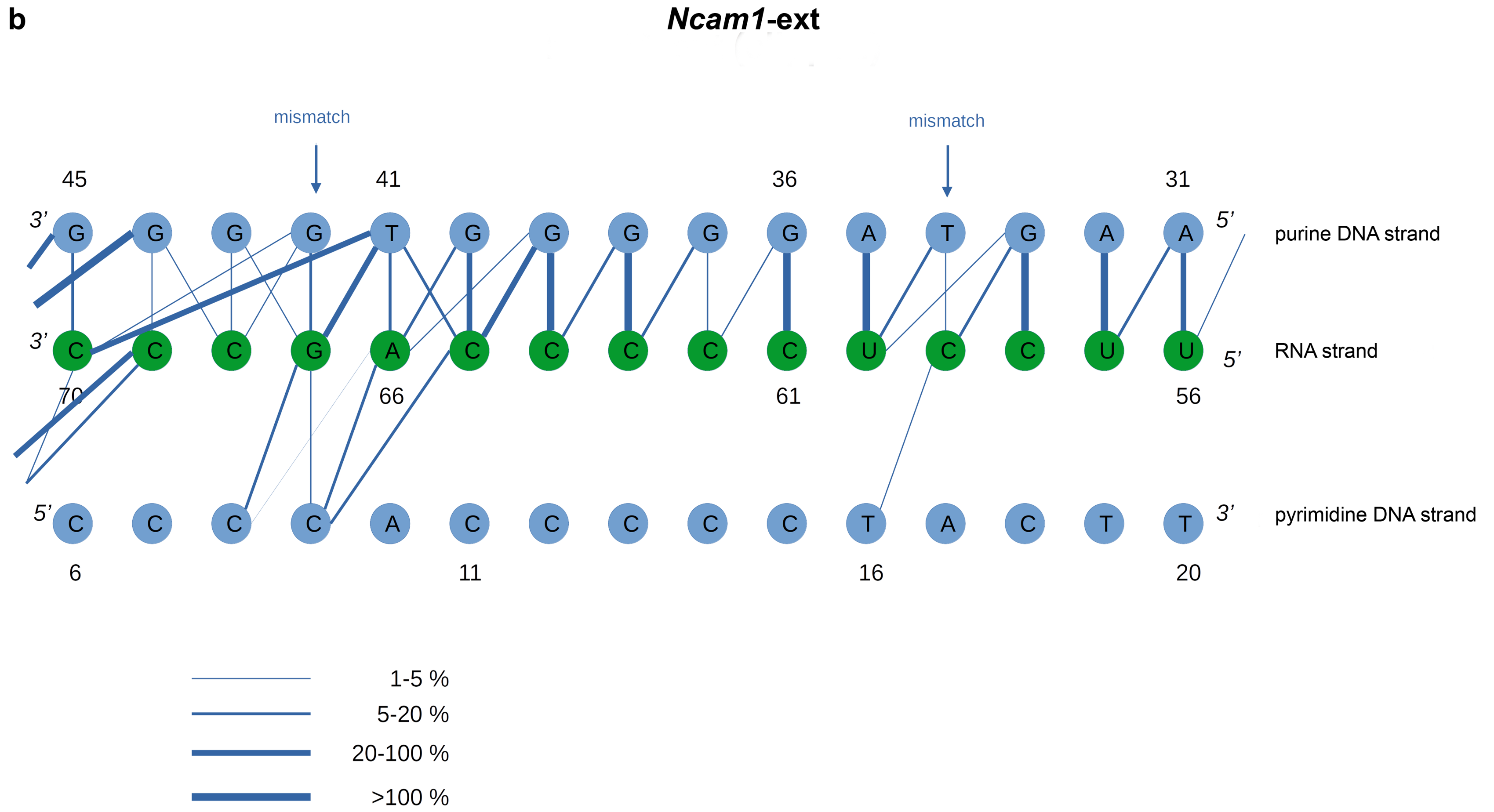

# Supplementary Figure 1
