# Supplementary material for: EphrinA5 regulates cell motility by modulating Snhg15/DNA triplex-dependent targeting of DNMT1 to the Ncam1 promoter
Source: Epigenetics Chromatin. 2023 Oct 26;16:42. doi: 10.1186/s13072-023-00516-4 (PMC10601256; doi:10.1186/s13072-023-00516-4)
Supplement: Supplementary file 6 — Additional file 6. Additional methods. [file 13072_2023_516_MOESM6_ESM.docx]

**Additional file 1:**

**Supplementary Figure legends and Methods**

**EphrinA5 regulates cell motility by modulating *Snhg15*/DNA triplex-dependent targeting of DNMT1 to the *Ncam1* promoter**

Can Bora Yildiz^1,2^, Tathagata Kundu^3^, Julia Gehrmann^4^, Jannis Koesling^1^, Amin Ravaei^1,5^, Philip Wolff^1^, Florian Kraft^6^, Tiago Maié^4^, Mira Jakovcevski^1^, Daniel Pensold^1^, Olav Zimmermann^3#^, Giulia Rossetti^3,7,8#^, Ivan G. Costa^4#^, Geraldine Zimmer-Bensch^1,2#^*****

**Figure S1:** **Western blots confirming the specificity of antibodies against DNMT1 and EZH2.** (a) Immunoprecipitations from N2a whole cell lysate with normal rabbit IgG (#12-370, Merck) and rabbit anti-DNMT1 (#70-201, BioAcademia) antibodies. Detection was performed with the mouse anti-DNMT1 antibody (1:250, # ab13537, abcam). (b) The EZH2 antibody (1:1000, #5246, Cell Signaling Technology) was used for detection on whole cell lysate from the murine medial ganglionic eminence (MGE). Protein sizes are indicated in kDa, identified through SERVA Protein standard III.

**Figure S2**: **CLIP revealed no interaction of *Snhg15* and EZH2 in CB cells.** RNA recovery for IgG and anti-EZH2 antibody CLIP samples in CB cells (N = 5 biological replicates). For all investigated amplicons, the recovery for EZH2 could not be statistically differentiated from the IgG-based pulldown. Whiskers of the box plots extend 1.5 times the interquartile range from the 25th and 75th percentiles (Tukey style) while outliers are represented by hollow dots. Significances were determined with two-tailed Student’s *t*-test. ctrl-Fc: control Fc, efnA5-Fc: ephrinA5-Fc, CB: cerebellar granule, CLIP: UV cross-linking and immunoprecipitation.

**Figure S3: Expression of *NCAM1*/*Ncam1* is implicated in low-grade glioma as well as the migration of CB cells, where it can be downregulated via RNA silencing.** (a) High expression levels of *NCAM1* are associated with increased patient survival in low-grade glioma. Survival analysis is based on clinical data and gene expression counts from tumor samples of lower grade glioma patients downloaded from BioPortal (<http://www.cbioportal.org/study/clinicalData?id=lgg_tcga>) and The Cancer Genome Atlas (TCGA), respectively. (b) Knockdown efficiency of the applied *Ncam1* siRNA (N = 3 biological replicates). (c) Quantitative analysis of migration distance of CB cells (n = 557 for ctrl siR + ctrl-Fc, n = 455 for ctrl siR + efnA5-Fc, n = 481 for Ncam1 siR + ctrl-Fc, n = 495 for Ncam1 siR + efnA5-Fc, N = 4 biological replicates). Significances were determined with log-rank (a), Wilcoxon-Mann-Whitney test (b) and one-way ANOVA (c). Significance levels: *p* value < 0.05 *; *p* value < 0.01 **; *p* value < 0.001 ***. ctrl: control. efnA5: ephrinA5. LGG: low-grade glioma. siR: siRNA.

**Figure S4: Migratory analysis of CB cells upon stimulation with ephrinA5-Fc and downregulation of *EphA2*.** The motility of CB cells was reduced upon stimulation with ephrinA5-Fc. (a) Temporal color-coded migratory distance over 20 h of imaging. The starting point of migration for each cell is shown in dark blue and the end point in white. (b) Quantitative analysis of average migratory speed (n = 268 for ctrl siR + ctrl-Fc, n = 244 for ctrl siR + efnA5-Fc, n = 273 for *EphA2* siR + ctrl-Fc, n = 238 for *EphA2* siR + efnA5-Fc, N = 3 biological replicates). (c) Knockdown efficiency of the applied *EphA2*-siRNA (N = 3 biological replicates). Significances were determined with one-way ANOVA (b) and Wilcoxon-Mann-Whitney test (c). Significance levels: *p* value < 0.05 *; *p* value < 0.01 **; *p* value < 0.001 ***. Scale bar: 100 μm. ctrl: control. efnA5: ephrinA5. siR: siRNA.

**Figure S5: The murine *Ncam1* locus shows bivalent regulation in primary cerebellar tissue and CB cells.** (a) The peaks depict publicly available ChIP-seq data for H3K27me3 and H3K4me3 in the murine *Ncam1* locus in cerebellar tissue from various developmental stages. The corresponding GEO accession numbers for the datasets from top to bottom are as follows: GSM1000143, GSE29184, GSM1000090, GSM769027. Genomic regions targeted by the primers are shown in turquoise. The promoter is shown in dark blue, candidate cis-regulatory elements in red, CpG islands in green, and putative *Snhg15* binding sites in pink. (b-d) Native ChIP using anti-DNMT1 and anti-H3K27me3 with qPCR analysis targeting the *Ncam1* promoter region normalized against the input material and IgG (N = 4 biological replicates). Stimulation of CB cells with ephrinA5-Fc does not alter the DNMT1 or H3K4me3 enrichment within the *Ncam1* locus (b-d). (e) Native ChIP using anti-DNMT1, anti-H3K27me3 and anti-H3K4me3 with qPCR analysis targeting the muscle differentiation gene *MyoD* normalized against the input material and IgG (N = 4 biological replicates). The *MyoD* locus is enriched with DNMT1 and the repressive histone mark H3K27me3 but lacks the permissive histone mark H3K4me3 in CB cells. The results were tested for statistical significance with a two-tailed Student’s t-test (b-e). CB: cerebellar granule. ctrl: control. E14.5: embryonic day 14.5. efnA5: ephrinA5. P0: post-natal day 0. W8: post-natal week 8.

**Figure S6: Native ChIP reveals no changes in DNMT1 association and histone methylation signatures within the proximity of a *cis*-regulatory element of *Adamts14* and the putative *Snhg15* binding sites.** (a) Genomic regions targeted by the primers are shown in turquoise. The promoter is shown in dark blue, candidate cis-regulatory elements in red, and putative *Snhg15* binding sites in pink. (b-c) ChIP-qPCR analysis with anti-DNMT1 and anti-H3K27me3 antibodies for the promoter region of *Adamts14* normalized against the input material and IgG (N = 3 biological replicates for *P1* (b), N = 4 biological replicates for *P2* (c)). Significances were determined with two-tailed Student’s *t*-test. Significance levels: *p* value < 0.05 *; *p* value < 0.01 **; *p* value < 0.001 ***. ctrl: control. efnA5: ephrinA5.

**Figure** **S7:** Root mean square deviation (RMSD) plot for *Ncam1*, *Ncam1*-ext, *Adamts14*-1 and *Adamts14*-2 taken over the entire trajectory of 600 ns for (a) the RNA strand of the triple helix and (b) the DNA double helix.

**Figure** **S8**: **Comparison of energy contributions between the two *Adamts14* models (*Adamts14*-1 and *Adamts14*-2).** The hydrogen bond energies between individual residues at the same base pair level (i) were calculated by taking the sum of Lennard-Jones (LJ) and Coulomb (CB) short range interaction energies. The cross energies were calculated for a base pair level (i) by considering the sum of LJ and CB short range interaction energies of (i)th residue in chain B with (i+1)th and (i-1)th residue in chain A and C. The stacking energies were calculated for a base pair step level by considering the sum of LJ and CB short range interaction energies of (i)th and (i+1)th residues in chain A, B and C. The total plot shows the sum of the contribution of individual energies at the (i)th base pair level. In all calculations the energy contributions involving terminal residues were not included to avoid discrepancy in the number of terms that contribute.

**Figure** **S9**: **Comparison of energy contributions between the two *Ncam1* models (*Ncam1* and *Ncam1*-ext).** The hydrogen bond energies between individual residues at the same base pair level (i) were calculated by taking the sum of Lennard-Jones (LJ) and Coulomb (CB) short range interaction energies. The cross energies were calculated for a base pair level (i) by considering the sum of LJ and CB short range interaction energies of (i)th residue in chain B with (i+1)th and (i-1)th residue in chain A and C. The stacking energies were calculated for a base pair step level by considering the sum of LJ and CB short range interaction energies of the (i)th and (i+1)th residue in chain A, B and C. The total plot shows the sum of the contribution of individual energies at the (i)th base pair level. In all calculations the energy contributions involving terminal residues were not included to avoid discrepancy in the number of terms that contribute.

**Figure** **S10:** Display of all hydrogen bonded interactions of the RNA that occur with frequency >1% in the (a) *Adamts14*-1 and (b) *Ncam1*-ext simulation. For clarity the purine DNA strand was drawn above, and the pyrimidine DNA strand below the RNA. H-bonds between the two DNA strands as well as within individual strands are omitted to focus on the H-bond patterns of the RNA strand. Occurrence is in “H-bond units”, i.e., an occurrence of >100% indicates that on average there exists more than one H-bond between the respective residues.

**Table S1:** Sequences of applied primer pairs in (RT-)qPCR experiments.

**Table S2:** Detailed sequences of the simulated systems (*Adamts14*-1, *Adamts14*-2*, Ncam1, Ncam1*-ext*)*. Pairing between DNA sequences (black) and RNA sequences (red) is predicted to form triple helixes for two binding sites in the *Adamts14* promoter and one binding site at the *Ncam1* promoter. 5’ and 3’ indicates the orientation of the DNA and RNA strands. “|” indicates base pairing following triple helix canonical code, while “*” indicates positions with a mismatch.

**Table S3.** Differentially methylated sites in CB cells treated with ephrinA5-Fc. Table lists all probes/sites (DMSs) with an adjusted *p* value ≤ 0.05 (adj. *p*val).

Output of the function minfi::dmpFinder() for identified differentially methylated sites in CB cells treated with ephrinA5-Fc. The minfi::dmpFinder() function models the methylation ß-value of the respective CpG for each phenotype (i.e. "efnA5" or "control") with linear regression model and then determines whether the two regression models differ significantly from each other by use of an F-test. The table shows the parameters of the fitted regression models and the results of the F-test for all probes/sites (DMSs) with an adjusted p value ≤ 0.05 (adj. pval).

**Table S4:** Table depicts all 19 protein-coding genes upregulated after 24 h ephrinA5-Fc treatment and with putative triplex target DNA sites (TTS) for *Snhg15* (RNA-seq; Pensold et al. 2021).

**Supplementary Methods:**

*Cultivation of N2a cells*

Neuro-2a (N2a) cells were cultured in DMEM (#31966021, Gibco) supplemented with 10% fetal bovine serum (FBS) at 37°C, 5% CO_2_ and 95% relative humidity. Upon thawing, the medium was additionally supplemented with 100 U/mL penicillin and 100 µg/mL streptomycin until the first passage. After reaching 75% confluency, the N2a cells were harvested by mechanical dissociation, pelletized, and stored at -80°C.

*Immunoprecipitation and Western blotting*

N2a cell pellets were lysed by adding lysis buffer (0.1 M Na_2_HPO_4_, 100 mM NaH_2_PO_4_, 5 mM EDTA, 2 mM MgCl_2_, 0.1% (v/v) CHAPS, 10 ng/µL Leupeptin, 1 mM PMSF) and repeatedly homogenizing the samples with a pellet pestle for 1 h on ice. After 15 minutes of centrifugation at 16,000×g and 4 °C, the supernatant (whole cell lysate) was collected, and the protein concentration was determined using the Qubit 4 fluorometer (Invitrogen). Agarose A beads were washed twice with IP buffer (20 mM HEPES, 0.1 mM EDTA, 50 mM KCl, 0.05% (v/v) CHAPS) and subsequently incubated for 1 h on ice with 3 µg rabbit anti-DNMT1 antibody (#70-201, BioAcademia) or normal rabbit IgG for control (#12-370, Merck). Empty binding sites were blocked by adding 45 µg bovine serum albumin (BSA) for 1 h on ice. After washing twice with the IP buffer, whole cell lysate containing 2 mg of proteins was added for overnight incubation at 4°C. The beads were washed six times before SDS buffer (200 mM Tris (pH 6.8), 10% (v/v) β-mercaptoethanol, 0.002% (v/v) bromophenolblue, 40% (v/v) glycerol, 4% SDS) was added and the samples were heated to 95°C for 10 minutes. The subsequent SDS-PAGE was performed using the SERVA BlueVertical™ PRiME™ electrophoresis chamber with Laemmli buffer (#42556, Serva) and a HSE neutral Gel (#43245, Serva) according to the manufacturer’s instructions. Proteins were transferred to a nitrocellulose membrane (#71224, Serva) in Towbin buffer (#42558, Serva) and a semi-dry blotter (VWR) following the manufacturer’s guidelines. The membrane was blocked using 1x BlueBlock solution (#42591, Serva) for 1 h before incubation with either mouse anti-DNMT1 (1:250 in Blue Block, #ab13537, abcam) or rabbit anti-EZH2 (1:1000 in BlueBlock, #5246, Cell Signaling Technology) overnight at 4°C. After washing three times in TBS-T buffer (25 mM Tris-HCl (pH 7.5), 137 mM NaCl, 2.7 mM KCl, 0.05% (v/v) Tween-20), the membrane was incubated for 1 h at room temperature with either the sheep anti-mouse (1:1000 in BlueBlock, #NA931, GE Healthcare) or donkey anti-rabbit (1:1000 in BlueBlock, #NA934, GE Healthcare) horseradish peroxidase (HRP)-conjugated secondary antibody. After three more washing steps in TBS-T, the chemiluminescence was elicited by adding HRP substrate solution (#42588, Serva). Imaging was performed using the ChemiDoc MP Imaging System (Bio-Rad).

*Survival Analysis*

The survival analysis was implemented in R. It was based on clinical data and gene expression counts from tumor samples of lower grade glioma patients downloaded from BioPortal (<http://www.cbioportal.org/study/clinicalData?id=lgg_tcga>) and The Cancer Genome Atlas (TCGA), respectively. To download the gene expression counts, we applied the R package TCGAbiolinks setting the parameters for GDCquery as follows: project=”TCGA-LGG”, data.category=”Transcriptome Profiling”, data.type=”Gene Expression Quantification”, workflow.type=“HTSeq – Counts” and sample.type=c(“Primary solid Tumor”, “Recurrent Solid Tumor”). The raw gene counts were normalized by applying DESeq2::varianceStabilizingTransformation(). Afterwards, the patients were divided into two groups (high expression, low expression) based on whether their NCAM1 expression level is above or below the median NCAM1 expression level among all patients. Using survival::Surv() and survival::survfit() while setting the time parameter to the survived months and the event parameter to the patients’ vital status from the clinical data a Kaplan-Meier curve object was created. It was plotted with survminer:: ggsurvplot() setting risk.table=TRUE. If not indicated otherwise, default parameters have been passed to the applied R functions.
